# Quality of life among patients with multiple sclerosis and voiding dysfunction: a cross-sectional study

**DOI:** 10.1186/s12894-020-00590-w

**Published:** 2020-06-03

**Authors:** Fatemeh Nazari, Vahid Shaygannejad, Mehrdad Mohammadi Sichani, Marjan Mansourian, Valiollah Hajhashemi

**Affiliations:** 1 Isfahan neurosciences Research center, Isfahan University of Medical Sciences, Isfahan, Iran; 2 Department of Adult Health Nursing, Faculty of Nursing and Midwifery, Isfahan University of Medical Sciences, Isfahan, Iran; 3 Department of Neurology, School of Medicine, Isfahan University of Medical Sciences, Isfahan, Iran; 4 Isfahan Kidney Transplantation Research Center, Isfahan University of Medical Sciences, Isfahan, Iran; 5 Department of Urology, School of Medicine, Isfahan University of Medical Sciences, Isfahan, Iran; 6 Department of Epidemiology & Biostatistics, School of Health, Isfahan University of Medical Sciences, Isfahan, Iran; 7 Isfahan Pharmaceutical Sciences Research Center, Isfahan University of Medical Sciences, Isfahan, Iran; 8 School of Pharmacy and Pharmaceutical Sciences, Isfahan University of Medical Sciences, Isfahan, Iran

**Keywords:** Multiple sclerosis, Urinary symptoms, Quality of life

## Abstract

**Background:**

Evaluating the usefulness of treatment requires a direct measurement of the health-related quality of life (QOL). Therefore, this study was carried out aiming to determine the QOL of patients with MS and voiding dysfunction.

**Methods:**

This cross-sectional study was carried out using multi-stage random cluster sampling method on 602 patients with MS in Isfahan, Iran. All data were collected through interviews using standard questionnaires including International Prostate Symptom Score (IPSS), and the Multiple Sclerosis Quality of Life-54 (MSQOL-54). Data were analyzed using descriptive and inferential statistical tests.

**Results:**

The prevalence rate of mixed, irritative, and obstructive urinary symptoms was 52.2, 25.5, and 6.5%, respectively. The mixed symptom had the highest prevalence among men and women with rates of 56.5 and 51.1%, respectively. The prevalence of irritative and obstructive symptoms was, respectively, higher and statistically significant among women alone and men alone (*P* <  0.05). The prevalence of irritative symptoms was higher among patients with MS, EDSS score ≤ 3, disease duration of less than 5 years, and with clinically isolated syndrome. In addition, the prevalence of mixed symptoms was higher among patients with MS of over 30 years of age with a Pre-high school degree, severe disability, disease duration of over 10 years, and progressive MS; the difference was statistically significant (*P* <  0.05). There was a difference in the combined dimensions of physical and mental health of QOL between the two groups with and without urinary symptoms (P <  0.05). Logistic regression analysis revealed that there was a higher probability of a urinary problems among patients with MS and high age [3.273 (1.083–9.860); *P* = 0.035].

**Conclusions:**

Mixed urinary symptoms are highly prevalent among MS patients and affect QOL dimensions. In order to improve QOL, more attention and focus should be paid to urinary problems in MS patients.

## Background

Multiple sclerosis (MS) is a chronic inflammatory demyelinating, and neurodegenerative disease with unpredictable course in young adults [1]. The onset of MS can occur at any age; however, it is often observed between the ages of 20 and 50 years, and its incidence rate is three times higher among women compared to men [2]. Today, around 2.5 million individuals suffer from MS around the world [3]. MS is the most common progressive neurological disorder, causing a wide range of functional limitations, including lower urinary tract symptoms (LUTS), which are observed in 50–80% of patients during the course of the illness and have a dramatic negative impact on patients’ quality of life (QOL) and their ability to execute social roles [4, 5]. Neurogenic LUTS in MS can include urinary incontinence (UI) or urinary retention or a combination of both [2, 6]. Over 90% of patients with MS show some LUTS 10 years after the onset of the disease [7]. LUTS differ to a large extent from one patient to the other [8] and are accompanied by significant psychological effects and the highest rate of socially debilitating outcomes of MS [9], which cause severe limitations in activity levels [6]. Patients with MS and bladder dysfunction reported that LUTS constrained their daily activities, and the physical and clinical burden associated with MS and UI negatively affected their QOL, causing feelings of shame, depression, skin fragility, and social isolation [10]. Moreover, LUTS limits their lifestyle choices, and they require facilities to facilitate their urination in the bathroom and become dependent on caregivers [11, 12]. Furthermore, this complication reduces their participation in health development programs, and hence, the secondary complications of the disease increase and their QOL is further reduced [13]. LUTS associated with MS significantly increase the economic consequences related to nursing care and incontinence therapies and related urinary infections [14]. The overall cost of care for bladder complications in the United States, including loss of productivity, has been estimated to be more than 65 billion dollars annually [15]. In addition, bladder dysfunction has a negative effect on the sexual performance of the patient and is a threat to the upper urinary tract as it can lead to permanent urinary tract disorder; thus, it has been recognized as a health problem among this population [15–17]. The results of a study revealed that the mean score of health-related QOL among patients with MS with overactive bladder symptoms was significantly lower in comparison to that of the control group [9]. In addition, it was reported in another study that the QOL scores of patients with MS with UI were significantly lower compared with patients with MS without UI in physical and mental performance dimensions [18]. LUTS even affects QOL scores, especially the general dimensions among men and sexual dimensions among women suffering from clinically isolated syndrome (CIS) [19]. Patients with MS have lower QOL in comparison to the general population, as well as compared to patients with other chronic illnesses. The extensive physical disability of, lack of effective treatment for, and unknown causes of MS are indicative of its negative impact on the QOL of patients [20]. A longitudinal study showed that the ratio of patients with MS and at least one symptom of bladder dysfunction significantly increased over time both among men and women and had a significant relationship with high levels of physical disability and health-related QOL at any point of follow-up for both men and women with MS. [21]

Several studies have shown that patients with MS, even those with relatively mild physical disabilities, have a significantly lower QOL compared with the general population. It has been shown that a remarkable decrease in QOL is associated with long duration of illness among patients with MS and bladder disorders, indicating the need to identify the signs and symptoms involved in their reduced QOL [22]. Therefore, due to differences in the incidence rate of bladder dysfunction in different communities, clinical symptoms and bladder disorder, and the demographic characteristics of patients with MS, and the serious impact of these disorders on QOL, the present study was conducted aiming to evaluate the QOL of patients with MS and voiding dysfunction.

## Methods

This cross-sectional study was carried out from 23 August 2017 to 19 April 2016 on 603 patients with MS who referred to the neurology clinic of Kashani and Alzahra treatment centers affiliated to Isfahan University of Medical Sciences, Isfahan, Iran. In this study, the participants were selected using the multi-stage random cluster sampling method and cluster volume was determined in accordance with their population size. The duration of the disease and gender were, respectively, considered as the first and second classes, and the separated number of men and women as clusters. In general, the sample was selected proportional to the sample size for men and women classes using a random number table. Based on the results of a previous study [23] with the prevalence of urinary symptoms (*P* = 0.51), 5% accuracy, and 95% confidence interval (CI) and taking into account d = 0.04 and a 10% sample loss, the number of samples was estimated to be 660. Among the selected subjects, 38 and 19 patients were excluded from the study due to their lack of willingness to cooperate and complete the questionnaire, and due to MS relapse and corticosteroid therapy, respectively.

The study inclusion criteria were a definitive diagnosis of MS by a neurologist based on the 2010 Revised McDonald Diagnostic Criteria [24], age of above 18 years, admittance to a referral clinic, physical and mental ability to answer questions (lack of physical or cognitive deficiencies causing inability to answer the questions correctly), residing in Isfahan, willingness to participate in the study, and lack of bladder dysfunction for a different cause (before the onset of MS). Moreover, the exclusion criteria included the unwillingness to continue collaboration in the study and complete the questionnaire, MS relapse in the previous month, and treatment with corticosteroids.

After completion of informed consent forms by the subjects, all the data in this study were collected through interviews using a four-part questionnaire. The first part of the questionnaire included 6 questions on demographic characteristics (age, gender, marital status, educational status, occupation, and economic status). The second part contained 5 questions regarding clinical information (duration of disease, age at the time of diagnosis, Expanded Disability Status Scale (EDSS) score, pattern of clinical course of MS, and type and duration of use of drugs). The third part included the International Prostate Symptom Score (IPSS). The IPSS includes 7 specific questions on irritative and obstructive symptoms and an additional question on general satisfaction with the urinary condition which has not been used in the scale scoring. The IPSS questionnaire has been recommended for the preliminary assessment of LUTS [25]. The scoring system of this questionnaire was based on patient statements and it was not an objective questionnaire. It includes questions about daily urine frequency, urgency, and nocturnal polyuria, reflecting the status of irritative or storage symptoms (maximum score of 15), and incomplete voiding of the bladder, interruption of the urine flow, poor flow of urine, and exerting force at the onset of urination indicating obstructive or voiding symptoms (maximum score of 20). The questions are scored based on a 6-point Likert scale ranging from 0 (no problem) to 5 (always), and the scoring of the symptoms was based on the frequency scale ranging from 0 to 5. The total score was calculated through summing up the quantitative score of the 7 questions on LUTS (range: 0–35); moreover, scores of 0–7, 8–19, and 20–35 indicate lack of symptoms or presence of mild symptoms, moderate symptoms, and severe symptoms, respectively. In this study, the Persian version of the IPSS questionnaire translated by Panahi et al. was used [26]. They showed that this questionnaire with a Cronbach’s alpha value of 0.7, intra-class correlation coefficient (ICC) of 0.87, and Pearson’s product moment coefficient of 0.92 is a valid and reliable scale. The fourth part of the questionnaire included the 54-item Multiple Sclerosis Quality of Life (MSQOL-54), of which 18 questions addressed 14 areas specific to patients with MS, including physical performance (10 questions), role limitation due to physical problems (4 questions), role limitation due to mental problems (3 questions), health changes (1 question), social function (3 questions), health threats (4 questions), sexual function (4 questions), satisfaction with sexual function (2 questions), pain (3 questions), energy (5 questions), perception of general health (5 questions), total QOL (2 questions), cognitive function (4 questions), mental well-being (5 questions), and 36 questions addressed general QOL. The questions were scored based on Likert scales with 2 to 7 options. Finally, the QOL score of the patients was determined by the scores considered for the two combined dimensions of physical and mental health. Each question is scored between 0 and 100. The two combined physical and mental health dimensions of QOL were obtained through calculating the specific weight percentage for each final score in each dimension. For all dimensions, higher scores represented a better status. In this study, the Persian version of the MSQOL-54 questionnaire was used which was standardized by Ghaem et al. [27] with a Cronbach’s alpha coefficient of 0.962 for the measurement of QOL among patients with MS; they reported that it had appropriate structural characteristics, validity, and reliability. Also physical disability was rated by Expanded Disability Status Scale (EDSS) that assess pyramidal, cerebellar, brainstem, sensory, bowel and bladder, visual and mental functions. EDSS was examined by neurologist with scoring between 0 and 10 [24]. According to Jones’ criteria, they are classified into three categories: mild (0–3), moderate (3.5–5.5), severe (more than 7) [28].

This scale was completed by the researcher under the supervision of a neurologist. The collected data were analyzed using descriptive statistical tests of mean and standard deviation (SD) and inferential statistical tests including chi-square (χ^2^) test, independent t-test, analysis of variance (ANOVA), and logistic regression in SPSS (version 18, SPSS Inc. Chicago, IL, USA). The significance level was considered to be less than 0.050.

## Results

This study included 602 patients with MS. 474 (78.7%) and 128 (21.3%) of the subjects were women, men, and 415 (68.9%), 149 (24.8%), and 38 (6.3%) of the subjects were married, single, and divorced and widowed respectively. Moreover, 244(40.5%), 214(35.5%), 145(24.0%) had university degrees, high school and Secondary school or below, respectively. 128(21.2%), 378(62.7%), 45(7.5%), 52(8.6%) were employed, housewife, retired and disabled, and unemployed, respectively. The mean and SD of the age of the participants, duration of MS, and disability score were 35.45 ± 9.08 years, 7.63 ± 5.56 years,, and 2.45 ± 2.11, respectively. Moreover, 16(2.7%), 173 (28.6%), 247 (41.0%), 167 (27.7%) were in the group below 20 years, 20–29 years,30–39 years, high school and 40–50 years., respectively. According to the EDSS, 432 (71.6%) patients had mild, 151(25.0%) patients had moderate, and 20 (3.4%) patients had severe disability. In addition, 459(76.1%), 116 (19.3%), and 28(4.6%) of the patients had a clinical relapsing-remitting course, progressive, and CIS, respectively.

Of the 602 patients with MS, mild, moderate, and severe urinary symptoms had a rate of 58.8, 32.6, and 6.8%, respectively. The prevalence rate of mixed, irritative, and obstructive symptoms was 52.2, 25.5, and 6.5% respectively. Moreover, 15.4% of women and 17.1% of men lacked any urinary symptoms. The prevalence rates of mixed, irritative, and obstructive symptoms were 51.1, 28.1, and 4.5% among women and 56.5, 16.3, and 10.1% among men, respectively. Chi-square test showed a statistically significant difference between the two groups in this respect (*P* = 0.020). The prevalence of urinary symptoms differed significantly between age groups of below or above 30 years and also among individuals with different educational levels. There was no significant difference among subjects with and without urinary symptoms in terms of marital status, and body mass index (BMI) (Table 1).

**Table 1:** The prevalence of symptoms of bladder dysfunction based on individual variables (sex, age group, marital status, education, and BMI)

Variables		Lack of LUTS	Irritative (storage) symptoms	Obstructive (voiding) symptoms	Mixed symptoms	P -values
Sex	Total	94 (15.6)	154 (25.6)	39 (6.5)	315 (52.3)	0.014
	Female	72 (15.2)	134 (28.3)	26 (5.5)	242 (51.1(	
	Male	22 (17.2)	20 (15.6)	13 (10.2)	73 (57.0)	
Age group (years)	< 30	44 (23.4)	47 (25.0)	10 (5.3)	87 (46.3)	0.004
	> 30	50 (12.1)	107 (25.8)	29 (7.0)	228 (55.1)	
Marital status	Single	35 (18.6)	40 (21.3)	9 (4.8)	104 (55.3)	0.155
	Married	59 (14.3)	114 (27.5)	30 (7.2)	211 (51.0)	
Education level	Pre-high school degree	12 (8.3)	36 (24.8)	5 (3.4)	92 (63.4)	0.002
	high school degree	29 (13.6)	63 (29.6)	16 (7.5)	105 (49.3)	
	College	53 (21.7)	55 (22.5)	18 (7.4)	118 (48.4)	
BMI (kg/m^2^)	< 18.5	5 (10.6)	12 (25.5)	2 (4.3)	28 (59.6)	0.777
	18.5–25	57 (17.4)	83 (25.4)	23 (7.0)	164 (50.2)	
	> 25	32 (14.0)	59 (25.9)	14 (6.1)	123 (53.9)	

*BMI* Body Image Index, Data was presented as frequency (percentage) for categorical variables. *P*-values were derived from chi-square test

Among the patients, 19.7, 4.6, and 15.0% of the individuals with, respectively, mild, moderate, and severe disability had no urinary symptoms. In addition, 44.0, 72.8, and 75.0% of the patients with, respectively, mild, moderate, and severe disability had concurrent irritative and obstructive symptoms, and chi-square test showed a significant difference among the three groups (*P* <  0.001). No urinary symptoms were observed among 19.5, 16.5, and 9.4% of patients with the illness duration of, respectively, less than 5 years, 5–10 years, and more than 10 years. Moreover, 43.3, 50.5, and 67.5% of the patients with the illness duration of, respectively, less than 5 years, 5–10 years, and more than 10 years had concurrent irritative and obstructive symptoms (mixed), and chi-square test indicated a significant difference among the three groups (*P* = 0.001). No urinary symptoms were observed among 32.1, 17.4, and 5.2% of the patients with, respectively, CIS, relapsing-remitting MS (RRMS), and progressive MS. Furthermore, 32.1% of the patients with CIS had only irritative symptoms, and 48.1 and 75.0% of the patients with, respectively, RRMS and progressive MS had simultaneous irritative and obstructive symptoms, and chi-square test showed two significant differences among the three groups (*P* <  0.001) (Table 2).

**Table 2:** The prevalence of symptoms of bladder dysfunction based on clinical variables (Disease duration, EDSS, and clinical course)

Variables		Lack of LUTS	Irritative symptoms	Obstructive symptoms	Mixed symptoms	*P*-values
EDSS Class	Mild	84 (19.5)	122 (28.3)	35 (8.1)	190 (44.1)	< 0.001
	Moderate	7 (4.6)	30 (19.9)	4 (2.6)	110 (72.8)	
	Severe	3 (15.0)	2 (10.0)	0.0 (0.0)	15 (75.0)	
Disease Duration (years)	< 5	45 (19.5)	70 (30.3)	16 (6.9)	100 (43.3)	0.001
	5–10	34 (16.1)	54 (25.6)	16 (7.5)	107 (50.7)	
	> 10	15 (9.4)	30 (18.8)	7 (4.4)	108 (67.5)	
clinical course	CIS	9 (32.1)	9 (32.1)	3 (10.7)	7 (25.0)	< 0.001
	RRMS	79 (17.2)	126 (27.5)	32 (7.0)	221 (48.3)	
	Progressive MS	6 (5.2)	19 (16.4)	4 (3.4)	87 (75.0)	

*LUTS* lower urinary tract symptoms, *EDSS* Expanded Disability Status Scale, *CIS* Clinically isolated syndrome, *RRMS* Relapsing-remitting multiple sclerosis. Data was presented as frequency (percentage) for categorical variables. *P*-values were derived from chi-square test

The mean score of all dimensions of QOL, except for health changes, as well as the combined dimensions of physical and mental health in the LUTS-free group was significantly higher compared to the group with LUTS. There was a statistically significant difference in all dimensions, except for health changes (Independent t test, *P* <  0.050), among the two groups (Table 3). Moreover, the mean score of all dimensions of QOL, except for health changes and satisfaction with sexual function, as well as the combined dimensions of physical and mental health in the mild group was significantly higher compared to the group with moderate and severe LUTS. There was a statistically significant difference in all dimensions, except for health changes and satisfaction with sexual function (ANOVA, P <  0.050), among the three groups (Table 4). Logistic regression analysis also suggested a higher possibility of urinary problems among patients with MS and a higher age [3.273 (1.083–9.860); *P* = 0.035] (Table 5).

**Table 3:** Comparison of the mean scores of quality of life domains and physical and mental health composite dimensions of quality of life between the two groups with and without LUTS

QOL domains	Without of LUTS	With LUTS	*P*-value
	Mean (SD)	Mean (SD)	
Physical function	76.80 (27.68)	55.70 (32.10)	< 0.001
Role limitations due to physical problems	64.89 (42.65)	37.65 (43.61)	< 0.001
Role limitations due to emotional problems	56.38 (46.89)	41.99 (46.29)	0.006
Pain	83.37 (20.99)	71.12 (25.95)	< 0.001
Emotional well-being	67.87 (18.02)	58.25 (20.49)	< 0.001
Energy	59.49 (18.62)	49.39 (20.72)	< 0.001
Health perceptions	63.51 (20.76)	53.75 (21.00)	< 0.001
Social function	82.35 (19.85)	71.54 (22.69)	< 0.001
Cognitive function	81.43 (20.95)	71.65 (26.05)	< 0.001
Health distress	76.28 (23.17)	66.59 (28.40)	< 0.001
Sexual function	80.21 (29.33)	63.18 (31.86)	< 0.001
Change in health	49.20 (28.74)	46.41 (29.47)	0.397
Satisfaction with sexual function	70.61 (31.38)	56.89 (34.43)	0.004
Overall QOL	72.67 (15.99)	62.37 (20.25)	< 0.001
Physical health composite score	71.09 (18.84)	57.58 (19.99)	< 0.001
Mental health composite score	69.19 (17.97)	58.26 (20.67)	< 0.001

*LUTS* lower urinary tract symptoms, *QOL* Quality of life; *P*-values were derived from Independent t Test

**Table 4:** Comparison of the mean scores of quality of life domains and physical and mental health composite dimensions of quality of life between the three groups with different severity levels of LUTS

QOL domains	Mild (*n* = 260)	Moderate (*n* = 197)	Severe (*n* = 51)	*P*-values
	Mean (SD)	Mean (SD)	Mean (SD)	
Physical function	65.50 (30.18)	47.56 (30.90)	37.16 (29.62)	< 0.001
Role limitations due to physical problems	50.09 (44.62)	26.14 (39.31)	18.63 (34.94)	< 0.001
Role limitations due to emotional problems	49.36 (46.63)	36.72 (45.22)	24.83 (41.01)	< 0.001
Pain	76.95 (23.19)	65.43 (27.04)	63.46 (28.51)	< 0.001
Emotional well-being	62.62 (19.40)	54.03 (21.07)	52.23 (19.02)	< 0.001
Energy	54.51 (20.29)	44.57 (19.58)	41.96 (20.79)	< 0.001
Health perceptions	58.25 (20.61)	49.44 (20.07)	47.45 (21.80)	< 0.001
Social function	79.17 (18.66)	65.36 (24.00)	56.53 (21.81)	< 0.001
Cognitive function	75.88 (24.54)	68.17 (26.98)	63.52 (26.37)	< 0.001
Health distress	73.44 (26.48)	60.07 (29.30)	56.86 (25.96)	< 0.001
Sexual function	70.02 (29.25)	55.96 (33.06)	55.55 (33.18)	< 0.001
Change in health	49.04 (29.37)	44.42 (29.57)	40.69 (28.70)	0.086
Satisfaction with sexual function	60.63 (32.06)	51.92 (36.91)	56.82 (34.95)	0.092
Overall QOL	67.21 (17.94)	58.24 (20.78)	53.66 (23.00)	< 0.001
Physical health composite score	65.15 (18.03)	50.12 (18.81)	47.04 (19.02)	< 0.001
Mental health composite score	63.77 (19.82)	53.60 (20.03)	48.26 (19.52)	< 0.001

*LUTS* lower urinary tract symptoms, *QOL* Quality of life, *SD* Standard deviation; *P*-values were derived from one-way ANOVA

**Table 5:** Multivariable logistic regression analysis of patients with multiple sclerosis and urinary problems

Characteristic		Unadjusted odds ratio (95% CI)	*P*-values	Adjusted odds ratio (95% CI)	*P*-values
Physical health composite dimensions of QOL		1.054 (1.032 to 1.077)	< 0.001	1.035 (1.000 to 1.071)	0.050
Mental health composite dimensions of QOL		1.035 (1.021 to 1.049)	< 0.001	1.005 (0.979 to 1.032)	0.695
Gender	Female	0.819 (0.463 to 1.449)	0.492	0.365 (0.130–1.029)	0.057
	Male	Ref			
Age (year)	< 30	2.660 (1.624to 4.357)	< 0.001	3.273 (1.083 to 9.860)	0.035
	> 30	Ref			
BMI (kg/m2)		0.958 (0.903 to 1.017)	0.159	0.941 (0.855 to 1.036)	0.215
Marital status		1.400 (0.845 to 2.320)	0.192	0.000 (0.000 to -)	0.999
Educational status	Secondary school degree or below	0.338 (0.159 to 0.718)	0.005	0.591 (0.188 to 1.859)	0.369
	High school degree	0.678 (0.396 to 1.161)	0.157	0.764 (0.337 to 1.728)	0.517
	University degree	Ref			
Disease duration (year)		0.922 (0.876 to 0.972)	0.002	1.078 (0.963 to 1.207)	0.191
EDSS		0.647 (0. 545 to 0. 768)	< 0.001	0.798 (0.562 to 1.133)	0.207
clinical course	RRMS	0.496 (0.203 to 1.214)	0.125	0.321 (0. 100 to1.030)	0.056
	Progressive	0.053 (0.010 to 0.271)	< 0.001	0.000 (0.000 to -)	0.997
	CIS	Ref			
Marriage duration (year)		0.947 (0.912 to 0.983)	0.005	0.986 (0.915 to 1.062)	0.710
Age at onset of disease (year)		0.979 (.948 to1.011)	0.187	1.063 (0.966 to 1.169)	0.209

*QOL* Quality of life, *EDSS* Expanded Disability Status Scale, *RRMS* Relapsing-remitting multiple sclerosis, *CIS* Clinically isolated syndrome

OR adjusted based on individual variables (age, sex, marital status, duration of marriage, educational status, and occupation) and clinical variables (age at onset of disease, disease duration, EDSS, course of the disease, anxiety, depression, and stress)

## Discussion

MS with focal demyelinating lesions at different levels of the central nervous system (CNS) leads to urinary dysfunction [29]. In the present study 41.3% of patients had moderate to severe urinary symptoms. This finding was similar to the findings of the study by Sammarco with 43% moderate to severe bladder dysfunction among patients with MS. [14] The high prevalence of LUTS suggests the complexity of neural control of the function of the bladder and urinary tract in health and the location and nature of the neural lesions determines the pattern of bladder dysfunction [30].

In the present study, more than half of the patients with MS had mixed urinary tract symptoms and the incidence of storage or irritative symptoms was higher in comparison to urination or obstructive symptoms. In the study carried out by Onal et al., the prevalence rate of mixed symptoms among patients with MS (70%) was higher than the irritative (25%) and obstructive symptoms (5%) alone [31]. In addition, in the cross-sectional study by Ojewola et al., storage or irritative symptoms with an prevalence rate of 48.2% were more common than urination and post-urination symptoms with rates of 36.8 and 29.9%, respectively, in patients with MS. [32] Reports from western countries have indicated that irritative symptoms (storage phase) are prominent symptoms of the urinary tract, while in eastern countries, the prevalence of obstructive symptoms (voiding phase) is higher than irritative symptoms [33].

In addition, in this study, more than half of the men and women reported mixed symptoms, and the prevalence rate of irritative symptoms among women alone and obstructive symptoms among men alone was higher and the difference was statistically significant. In this regard, the study by Sand et al. also indicated that 60% of men and 50% of women with MS had reported mixed symptoms [23]; however, the results of the study by Aharony indicated that there was no significant correlation between the overall incidence of LUTS and gender [34]. The results of an investigation on 8284 men and women in China, Taiwan, and South Korea revealed that the prevalence of storage symptoms alone was higher among women compared to men (23.8% versus 12.6%, respectively) and obstructive symptoms alone had a rate of 7.2% [35]. However, due to the widespread nature of the lesions of the nervous system among patients with MS, numerous levels of control of the function of the bladder and intestine are observed [6], and the nature of urination complaints and LUTS varies among patients with MS. [36] In addition, LUTS change over time along with the dynamic course of MS. Therefore, continuous and regular follow-up assessment is required in patients with MS. [30]

In the current study, the prevalence of mixed urinary symptoms in patients over 30 years of age (55.1%) was higher than the age group of below 30 years (46%), and the difference was statistically significant. The results of a study showed a high prevalence rate for LUTS among men and women with a minimum age of 40 years and a significant increase in LUTS with age in the general population (at the age of 40–44 years to over 60 years with a prevalence of 49.9 and 69.7%, respectively) [35]. Based on a cross-sectional population-based study, there was a relationship between LUTS and age among women, but not among men, as younger men had a lower prevalence of LUTS in comparison to younger women, and older men had higher LUTS prevalence rate in comparison to older women [37]. Moreover, in two studies, a significant positive correlation was found between age and LUTS [38, 39].

In the current study, the prevalence of LUTS among patients with an education level of below diploma was higher than those with diploma and university degrees, and the difference was statistically significant. In this regard, the results of a cross-sectional study based on population in Australia did not show a significant positive correlation between LUTS and educational status [38].

The present study showed that the prevalence of irritative LUTS was higher in patients with MS with mild disability (EDSS ≤3). Nevertheless, the prevalence of mixed LUTS among patients with severe disability (EDSS ≥7) was higher than patients with mild to moderate disability and the difference was statistically significant. In the study by Di Filippo, 44% of patients with mild EDSS reported bladder dysfunction and this rate was increased among patients with moderate and severe disability (81%) and EDSS and the severity of corticospinal pathways had a significant correlation with the prevalence of irritative symptoms [40]. In another study, there was a positive relationship between EDSS and overactive bladder system score (OABSS) [38]; in addition, another study revealed that there was relationship between a high degree of disability and high levels of LUTS [41]. Khalaf et al. found that patients with MS with higher degree of disability had higher irritative symptoms (urgency and UI) [42], and the findings of the study by Onal revealed a weak correlation between EDSS severity and storage, urination, and total scores [31]. Arhrony found that irritative (storage) symptoms are associated with EDSS and the involvement of pyramidal pathways, but this association is very weak with obstructive symptoms [34]. Furthermore, another study revealed that the scores of irritative and obstructive symptoms were significantly increased with EDSS scores; however, the correlation coefficient for the scores of irritative symptoms (r = 0.60, *P* <  0.0001) was higher than obstructive symptoms [33]. In some other studies, there was a direct correlation between the EDSS score and irritative or obstructive symptom score [43, 44], albeit no relationship was reported between these variables in the studies by Miller and Porru [45, 46]. It seems that the differences in the results of different studies are due to the clinical course of MS which the patients under study were undertaking during the study period. De Carvalho showed that in patients with neuromyelitis optica spectrum disorder (NMO-SD), the severity of disability was a predictor of bladder dysfunction and detrusor-external sphincter dyssynergia (DESD) [47].

The present study indicated that the prevalence of irritative LUTS was higher among patients with MS and disease duration of less than 5 years, but the prevalence of mixed LUTS in patients with MS and disease duration of more than 10 years was higher compared to patients with MS and disease duration of less than 5 years and between 5 and 10 years, and the difference was statistically significant. The results of a study in Brazil showed a weak significant relationship between the duration of the disease and the presence of urinary dysfunction, and the degree of urinary dysfunction increased over the years [48]. In another study, the duration of disease and higher degree of disability only had a significant relationship with higher levels of urinary symptoms in women [14]. The review of data from the American Research Committee on Multiple Sclerosis (NARCOMS) showed that, with an increase in the duration of disease, the severity and prevalence of LUTS increased, so that on average 35–39% of patients 5–6 years after the onset of the disease, and in contrast, 64% of patients with a 17.1 year history of disease reported LUTS [49].

The current study revealed that the prevalence of LUTS was higher in patients with CIS; however, the prevalence of mixed LUTS in patients with progressive MS was higher in comparison to patients with MS with RRMS and CIS, and the difference was statistically significant. De Almedia reported prevalence rates of 63.5 and 100%, respectively, among patients with RRMS and primary progressive MS. [48] Moreover, a study by Wang et al. showed the lowest OBASS score in patients with CIS [29].

In the present study, patients with MS and LUTS symptoms compared to LUTS-free patients had lower QOL scores in all dimensions except for health changes and there was a significant difference in the combined physical and mental health dimensions of QOL between the two groups with and without LUTS. These results were basically similar to the results of a population-based cross-sectional study in Korea performed on 658 individuals; nearly a quarter (25.5%) of the population with LUTS had lower QOL scores [32]. Furthermore, the study by Khalaf suggested that patients with MS with immediate urgency and UI had significantly lower QOL in comparison to patients with MS without these symptoms [50].

In addition, there was a higher possibility of urinary problems in patients with MS with a higher age. In this regard, the studies by Jung2015 and McGrother2006 suggested that age can determine numerous and various bladder dysfunctions [51, 52]. Yet, Koldewijn1995 found that age is not an influencing factor on clinical or urodynamic profile [53]. It could be thinkable that age and MS, by means of potentiation of pathophysiological factors, may increase rate and /or severity of urinary problems in this specific population [54].

The strengths of this study included the appropriate sample volume and suitable ratio of men to women based on the proportion of MS prevalence rate in society. Considering the fact that the research was based on patients’ own statements on LUTS, the memory capacity of the subjects to recall their past information was one of the limitations in this study. Another possible limitation of the present study is the patients’ subjective perception of the investigated LUTS and the lack of objective urodynamic assessments and not necessarily at the time of study participation, thus not being representative for the investigated population.

## Conclusion

Mixed urinary symptoms are highly prevalent among MS patients and affect QOL dimensions. The chance of having a urinary disorder was higher among patients with MS and high age. In order to improve QOL, more attention and focus should be paid to urinary problems in MS patients.

## Data Availability

Not applicable.

## Abbreviations

BMI: Body mass index
CIS: Clinically isolated syndrome
DESD: Detrusor-external sphincter dyssynergia
EDSS: Expanded Disability Status Scale
IPSS: International Prostate Symptom Score
LUTS: Lower urinary tract symptoms
MS: Multiple sclerosis
NARCOMS: American Research Committee on Multiple Sclerosis
NMO-SD: Neuromyelitis optica spectrum disorder
OBASS: Overactive bladder system score
QOL: Quality of life
QOL: Quality of life
RRMS: Relapsing-remitting MS
SPMS: Secondary progressive MS
UI: Urinary incontinence
